# Outcome of transcatheter edge-to-edge mitral valve repair in patients with diabetes mellitus: Results from a real-world cohort

**DOI:** 10.1371/journal.pone.0276019

**Published:** 2022-10-17

**Authors:** Annemarie Kirschfink, Mhd Nawar Alachkar, Anas Alnaimi, Felix Vogt, Joerg Schroeder, Michael Lehrke, Michael Frick, Sebastian Reith, Nikolaus Marx, Mohammad Almalla, Ertunc Altiok

**Affiliations:** 1 RWTH University Hospital Aachen, Internal Medicine I, Cardiology, Angiology and Intensive Care Medicine, Aachen, Germany; 2 Clinic for Cardiology and Angiology, REGIOMED Hospital Coburg, Coburg, Germany; 3 Clinic for Cardiology, Angiology and Electrophysiology, St. Franziskus-Hospital, Münster, Germany; Albert Einstein College of Medicine, UNITED STATES

## Abstract

**Background:**

There are only limited data on patients with diabetes undergoing transcatheter edge-to-edge repair (TEER) in real-world settings. Previous data indicated patients with diabetes to have a worse prognosis. This study sought to evaluate safety and efficacy of TEER in patients with diabetes in a real-world cohort.

**Methods:**

In this monocentric study 340 consecutive patients with severe primary and secondary mitral regurgitation (MR) undergoing TEER were included. Immediate results of the procedure, intrahospital and one-year outcome were compared between patients with and without diabetes.

**Results:**

Diabetes was present in 109 patients (32%). Patients with diabetes were younger (77 y (71, 81) vs. 79 y (74, 83); p = 0.003), had more often ischemic cardiomyopathy (68% vs. 48%; p<0.001), previous coronary-artery bypass graft (35% vs. 20%; p = 0.002) and arterial hypertension (89% vs. 75%; p<0.001) compared to those without diabetes. Baseline NYHA class, type of MR (primary vs. secondary), left ventricular dimensions and function (ejection fraction: 37% (28, 50) vs. 40% (29, 55); p = 0.10) as well as severity of MR were not different between both groups. Success of the procedure (95% vs. 95%; p = 0.84), intrahospital mortality (5.5% vs. 4.8%; p = 0.98) and one-year follow-up regarding all-cause mortality (24.2% vs. 23.0%; p = 0.72), hospitalization for heart failure (37.4% vs. 31.0%, p = 0.23), NYHA class (p = 0.14) or MR severity (p = 0.59) did not differ between both groups.

**Conclusion:**

Our real-world data suggest that TEER seems to be similarly safe and effective in patients with severe MR and diabetes compared to those without diabetes.

## Introduction

Transcatheter edge-to-edge repair (TEER) has become a well-established and safe treatment option for selected patients with symptomatic severe mitral regurgitation (MR) and high surgical risk [1]. Different randomized controlled clinical trials with diverging inclusion and exclusion criteria lead to contradictory results concerning the efficacy of TEER using the MitraClip^TM^ device (Abbott Vascular Structural Heart, Menlo Park, CA, USA) [2, 3]. In the MITRA-FR and COAPT trial, 29% and 37% of all included patients had diabetes, respectively [3, 4]. Like previous studies on mitral valve surgery, analysis of the COAPT trial indicated that patients with diabetes have higher mortality and a trend to higher rates of hospitalization for heart failure (HHF) than those without diabetes [4–6]. In contrast, a previous single-center study showed no difference in the 3-months short-term follow-up after TEER regarding the improvement of functional status or mortality in patients with and without diabetes in a small cohort [7]. Regarding the controversial results, one has to take into consideration the strict inclusion criteria of the COAPT trial: in a recent single-center study could be demonstrated that just half of the real-world cohort fulfilled the COAPT criteria [8]. Therefore, it remains unclear whether patients with diabetes and severe MR benefit to a similar extent from TEER compared to non-diabetic subjects.

The objective of this study was to evaluate safety and efficacy of TEER in patients with diabetes mellitus in comparison to non-diabetics in a real-world cohort.

## Methods

### Patients’ selection

In this single-center study at the University Hospital of RWTH Aachen (Germany) all patients were retrospectively included, who were scheduled for TEER using the MitraClip^TM^ device between 2014 and 2019 and who presented with symptomatic severe primary or secondary MR according to the 2012 and 2017 published guidelines of the European Society of Cardiology [9, 10]. In advance, all 340 patients were evaluated by a multidisciplinary team, which confirmed TEER because of a high surgical risk. Factors like patients´ age, comorbidities, left ventricular ejection fraction, expectancy of life, logistic EuroSCORE, previous cardiac surgery, excessive calcification of the ascending thoracic aorta (“porcelain aorta”) or chronic pulmonary disease were taken into consideration for evaluating the individual surgical risk of each patient. Patients were classified by the presence of diabetes according to medical records. 2-dimensional (2D) transthoracic echocardiography (TTE) was performed at baseline, before discharge and during follow-up after one year. 3D transesophageal echocardiography (TEE) was carried out before and during the TEER procedure. **Table 1** shows all baseline clinical and echocardiographic characteristics. The protocol of this retrospective study was approved by the local ethics committee (EK 396/20). Patients provided informed written consent to have data from their medical records used for research.

**Table 1:** Clinical characteristics and echocardiographic parameters of the study population differentiated between patients without and with diabetes mellitus.

	No diabetes mellitus (n = 231)	Diabetes mellitus (n = 109)	p-value
***Clinical characteristics***			
**Age, years**	79 (74, 83)	77 (71, 81)	**0.003**
**Male, n (%)**	145 (62.8)	73 (67.0)	0.46
**Primary MR, n (%)**	56 (24.2)	21 (19.2)	0.38
**ICM, n (%)**	110 (47.6)	74 (67.9)	**<0.001**
**Previous CABG, n (%)**	45 (19.5)	38 (34.9)	**0.002**
**Previous PCI, n (%)**	109 (47.2)	61(56.0)	0.10
**HbA1c, %**	5.6 (5.2, 5.9)	6.8 (6.3, 7.9)	**<0.001**
**HTN, n (%)**	172 (74.5)	97 (89.0)	**<0.001**
**Atrial fibrillation, n (%)**	166 (71.9)	74 (67.9)	0.76
**eGFR<60 ml/min, n (%)**	148 (64.1)	78 (71.6)	0.10
**Dialysis, n (%)**	12 (5.2)	6 (5.5)	0.93
**PAD, n (%)**	93 (40.3)	40 (36.7)	0.73
**Log. EuroSCORE, %**	23 (15, 30)	24 (15, 35)	0.36
**NYHA class**			0.05
**I, n (%)**	2 (0.9)	0 (0)	
**II, n (%)**	25 (10.8)	16 (14.7)	
**III, n (%)**	155 (67.1)	58 (53.2)	
**IV, n (%)**	49 (21.2)	35 (32.1)	
**COAPT criteria fulfilled, n (%)**	70 (30.3)	39 (35.8)	0.27
***Imaging characteristics***			
**LVEF, %**	40 (29, 55)	37 (28, 50)	0.10
**LVEDD, mm**	56 (50, 63)	59 (51, 65)	0.16
**LVESD, mm**	44 (37, 54)	48 (35, 57)	0.33
**EROA, mm^2^**	30 (24, 41)	32 (24, 41)	0.40
**RVOL, ml**	49 (37, 62)	53 (40, 62)	0.71
**LA area, cm^2^**	29 (23, 34)	28 (23, 35)	0.67
**RA area, cm^2^**	23 (18, 28)	22 (18, 26)	0.68
**TR**			0.08
**Mild, n (%)**	116 (50.2)	50 (45.9)	
**Moderate, n (%)**	94 (40.7)	40 (36.7)	
**Severe, n (%)**	21 (9.1)	19 (17.4)	
**sPAP**	43 (34, 55)	42 (35, 53)	0.91

MR: mitral regurgitation, ICM: ischemic cardiomyopathy, CABG: coronary artery bypass graft, PCI: percutaneous coronary intervention, DM: diabetes mellitus, HTN: arterial hypertension, eGFR: estimated glomerular filtration rate, PAD: peripheral artery disease, COPD: chronic obstructive pulmonary disease, NYHA: New York Heart Association, LVEDD: left ventricular end diastolic diameter, LVEF: left ventricular ejection fraction, LVESD: left ventricular end systolic diameter, EROA: effective regurgitant orifice area, RVOL: regurgitant volume, LA: left atrium, RA: right atrium, TR: tricuspid regurgitation, sPAP: systolic pulmonary artery pressure.

### Follow-up and definition of outcome

Immediate results of the procedure, intrahospital as well as one-year outcome were compared between patients with and without diabetes. Intrahospital outcome consisted of procedural success (successful implantation of at least one clip and reduction of severity of MR by at least one grade), vascular complications (arteriovenous fistula, pseudo aneurysm or relevant bleeding requiring surgery or intervention) and intrahospital mortality.

One-year outcome comprised all-cause mortality, rehospitalization due to acute heart failure, NYHA dyspnea class and severity of MR in patients, who had available echocardiographic follow-up.

Follow-up data was gathered retrospectively from patient´s medical records in our patient data management system or by interviewing the patients or their primary care physicians per telephone about NYHA dyspnea class, need of hospitalization, cause of hospitalization during the first year and date of death.

### Statistical analysis

Continuous variables were expressed as mean (± standard deviation) or median (25th-75th percentile) and binary variables were expressed as count (percentage). Patients were classified according to the presence or absence of diabetes. Kolmogorov-Smirnov test for normal distribution rejected normality distribution (p<0.001). Continuous variables were compared with Mann-Whitney test and categorical variables were evaluated by chi-square test. Statistical analyses were performed with SPSS version 25.0 (IBM Corp., Armonk, NY, USA) and MedCalc version 13.0.0.0 (MedCalc Software Ltd., Ostend, Belgium). Statistical significance was awarded by p<0.05.

## Results

### Patients’ characteristics

340 consecutive patients who underwent TEER were included. Diabetes was present in 109 patients (32%) of the study group. Patients with diabetes were younger (77 y (71, 81) vs. 79 y (74, 83) years; p = 0.003), had more often ischemic cardiomyopathy (68% vs. 48%; p<0.001), previous coronary-artery bypass graft (35% vs. 20%; p = 0.002) and arterial hypertension (89% vs. 75%; p<0.001) compared to those without diabetes. All other baseline clinical and imaging characteristics including NYHA class, type of MR (primary vs. secondary), left ventricular (LV) dimensions and function (ejection fraction (EF): 37% (28, 50) vs. 40% (29, 55); p = 0.10) as well as severity of MR did not differ between both groups. **Table 1** summarizes patients’ clinical characteristics and their echocardiographic parameters.

### Intrahospital outcome

TEER was successfully performed in 324 patients (95%) with implantation of at least one clip and reduction of MR to moderate or less than moderate. Success of the procedure was similar between patients with and without diabetes (95% vs. 95%; p = 0.84) as was the number of implanted clips (1.4±0.6 vs. 1.5±0.7; p = 0.30), and the postprocedural mean transmitral pressure gradient (3.0 mmHg (2.6, 5.0) vs. 3.5 mmHg (2.7, 4.5); p = 0.96). Vascular complications were rare and comparable between both groups (2.8% in patients with diabetes diabetics and 3% in patients without diabetes; p = 0.84) There was no difference in intrahospital mortality between both groups (5.5% vs. 4.8%; p = 0.98) (**Table 2**).

**Table 2:** Intrahospital and one-year outcome of TEER differentiated between patients without and with diabetes mellitus.

	***Intrahospital outcome***		
	No diabetes mellitus (n = 231)	Diabetes mellitus (n = 109)	p-value
**Success of procedure, n (%)**	220 (95.2)	104 (95.5)	0.84
**Vascular complications, n (%)**	7 (3.0)	3 (2.8)	0.84
**Intrahospital mortality, n (%)**	11 (4.8)	6 (5.5)	0.98
**Number of clips**			0.30
**0, n (%)**	5 (2.2)	6 (5.5)	
**1, n (%)**	116 (50.2)	55 (50.5)	
**2, n (%)**	96 (42.6)	45 (41.3)	
**3, n (%)**	10 (4.3)	3 (2.8)	
**4, n (%)**	4 (1.7)	0 (0)	
**Pmean, mmHg**	3.5 (2.7, 4.5)	3.0 (2.6, 5.0)	0.96
			
			
	***One-year outcome*** *		
	No diabetes mellitus (n = 213)	Diabetes mellitus (n = 99)	p-value
**NYHA class**			0.14
**I, n (%)**	32 (18.2)	18 (23.1)	
**II, n (%)**	91 (51.7)	29 (37.2)	
**III, n (%)**	36 (20.5)	24 (30.8)	
**IV, n (%)**	17 (9.7)	7 (9.0)	
			
**Hospitalization due to HF, n (%)**	66 (31.0)	37 (37.4)	0.23
**One-year Mortality, n (%)**	49 (23.0)	24 (24.2)	0.72
			
**MR severity^≠^**			0.59
**Mild, n (%)**	89 (56.0)	42 (61.8)	
**Moderate, n (%)**	58 (36.5)	20 (29.4)	
**Severe, n (%)**	12 (7.5)	6 (8.8)	
			

* Patients with unsuccessful procedure or who did not survive to discharge were excluded from the follow-up analysis.

^≠^ Echocardiographic follow-up was available for 227 patients.

HF: heart failure, MR: mitral regurgitation, NYHA: New York Heart Association, Pmean: mean transmitral pressure gradient after TEER, TEER: transcatheter edge-to-edge repair.

### One-year outcome

**Fig 1:**
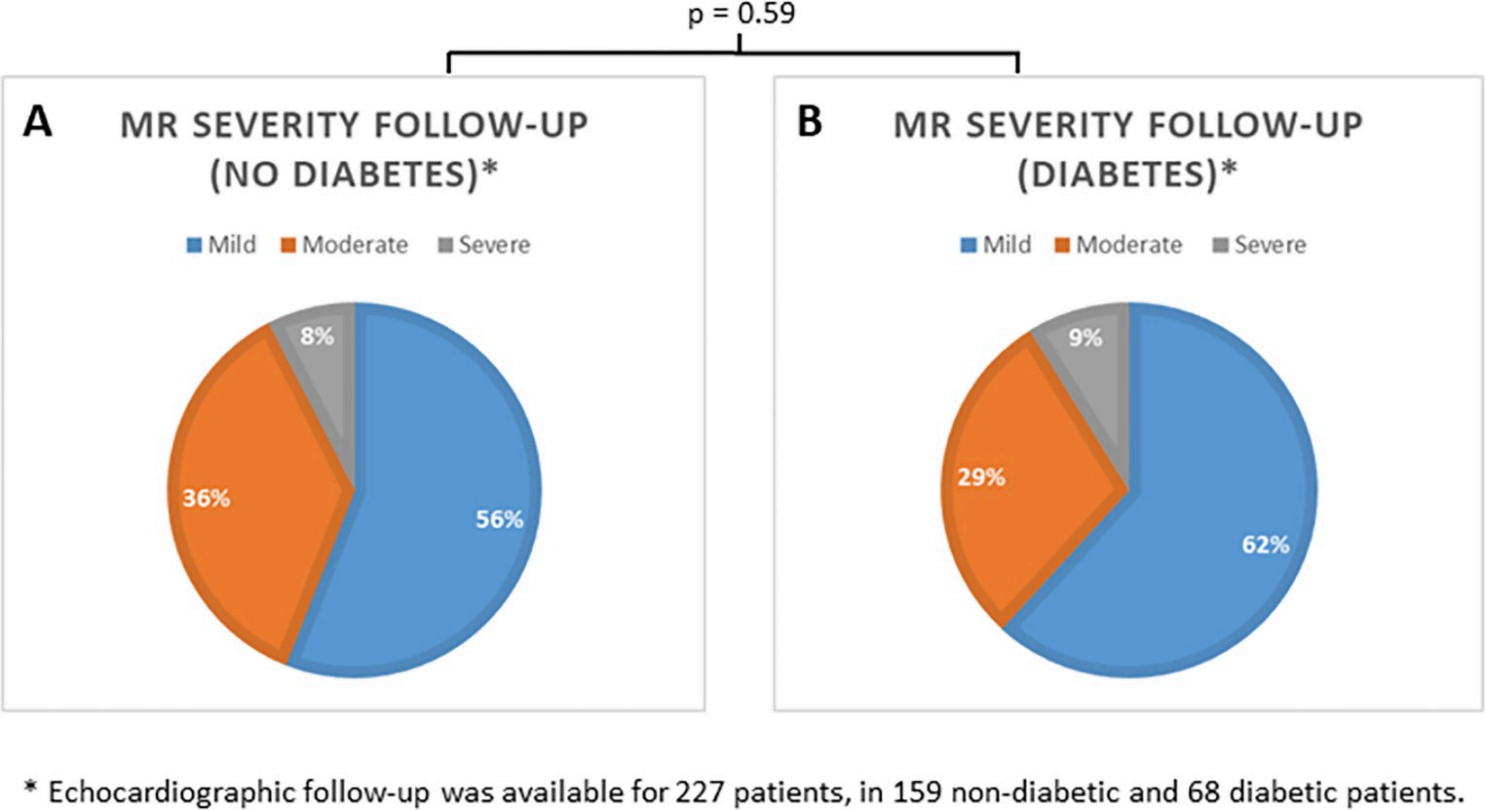
Mitral regurgitation (MR) severity grade at one-year follow-up. The left panel (A) shows MR severity grade in patients without diabetes and the right panel (B) in patients with diabetes mellitus.

At one-year follow-up, echocardiographic follow-up was available in 227 (73%) patients: MR severity did not differ between both groups (p = 0.59) (**Fig 1**).

**Fig 2:**
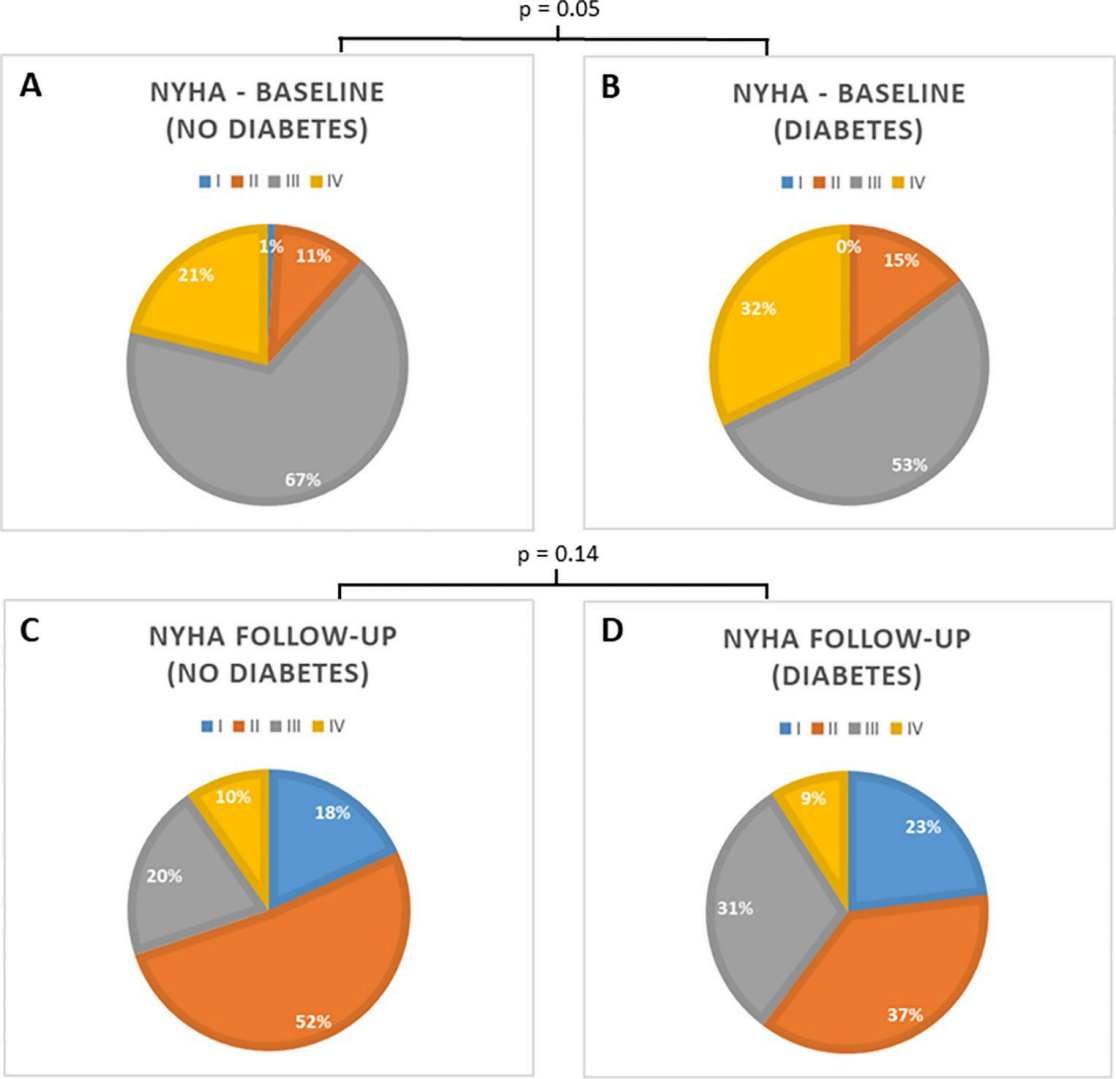
New York Heart Association (NYHA) class at baseline and at one-year follow-up. The upper panels (A and B) show NYHA class at baseline and the lower panels (C and D) at one-year follow-up in patients without (A and C) und with diabetes mellitus (B and D).

All-cause mortality (24% vs. 23%; p = 0.72) did not differ between groups. Hospitalization due to decompensated heart failure was similar between patients with and without diabetes (37% vs. 31%, p = 0.23). New York Heart Association (NYHA) class was also similar in both groups at one-year follow-up (p = 0.14) (**Fig 2**).

## Discussion

The major findings of this study were:

1. In this real-world cohort patients with diabetes, who underwent TEER because of severe symptomatic MR with high surgical risk, were younger and had more comorbidities.
2. TEER was nevertheless similarly safe and effective in patients with diabetes in comparison to patients without diabetes with no difference in success rate and intrahospital mortality.
3. At one-year follow-up, groups did not differ with respect to all-cause mortality, hospitalization due to heart failure, NYHA class or MR severity between both groups.

This is to our knowledge one of the largest real-world studies to investigate the effect of diabetes on safety and efficacy in TEER in a real-world cohort. Previous studies on mitral valve surgery showed that patients with diabetes exhibit a higher mortality than those without diabetes [5, 6]. Moreover, in patients with diabetes HHF was more frequent [11]. The recently published study regarding diabetes in COAPT showed, that patients with diabetes and severe secondary MR had a worse outcome than those without diabetes: the 2-year rates of death and the rate of HF hospitalization were higher in patients with diabetes. Like in our study, patients with diabetes were younger and had more comorbidities, whereas NYHA class, LV-EF and MR were not different between patients with and without diabetes. In comparison to guideline-directed medical therapy (GDMT) alone TEER reduced the outcomes of death and HHF and improved quality of life as well as exercise capacity in patients with diabetes. Therefore, the authors concluded, that the presence of diabetes should not prevent from TEER [4]. The different results according to outcome between the COAPT study and our study may be explained by even more diseased patients in the diabetes cohort with more comorbidities and higher systolic pulmonary artery pressure (sPAP) in the COAPT study. In our study, 70% of the patient without diabetes and 64% with diabetes did not fulfill the COAPT criteria EROA ≥ 30 mm^2^ and/or RV > 45 ml, LV-end-systolic diameter ≤ 70mm, LV-EF ≥ 20% and ≤ 50%, NYHA class II-IV, sPAP < 70 mmHg. A previous single center study could demonstrate that patient selection according to the COAPT criteria does not meet a real-world population: just half of the patients with severe secondary MR, who underwent TEER, fulfilled COAPT inclusion criteria. COAPT-like patients showed a better long-term clinical outcome, whereas the mid-term functional benefit was similar for all patients. Therefore, the authors conclude, that it is difficult to generalize the COAPT trial regarding a real-world setting [8].

The strict in- and exclusion criteria may also be the reason of the lower intrahospital mortality in patients with and without diabetes in the COAPT trial (2% vs 1%; p = 0.51) [4]. In our study intrahospital mortality was higher (5.5% vs. 4.8%; p = 0.98), but can be explained by more patients with poor prognosis, who died intrahospitally and would have been excluded according to the exclusion criteria of the COAPT trial: among others TEER was performed in 4 of 17 intrahospital deceased patients (25%) in cardiogenic shock, 1 patient (6%) after aortic valve replacement and 1 patient (6%) after myocardial infarction within the prior 30 days [2, 4].

Different real-world cohorts exist with divergent results regarding the impact of diabetes on safety and efficacy of TEER: Kaneko et al. (2017) reported that patients with secondary MR and diabetes were more often NT-proBNP nonresponders and therefore concluded that patients with diabetes were nonresponders to TEER using the MitraClip device. However, NT-proBNP nonresponders showed an improvement in NYHA class after 6 months (whereas data about HHF are lacking) [12]. Paulus et al. (2019) showed that patients with diabetes had a less functional outcome four weeks after TEER [13]. In contrast to the previous mentioned studies, Hellhammer et al. (2014) showed no difference in the 3-month short-term follow-up after TEER regarding the improvement of functional status or mortality in patients with and without diabetes [7]. The different results of these single-center studies may be explained by a small sample size and a relatively short follow-up.

In our study, in the diabetic group the mean HbA1c was 6.8% (6.3, 7.9), what shows a good control of diabetes in that group and may in part explain the similar outcome of patients with and without diabetes. Data on HbA1c in the COAPT trial are lacking, but Hellhammer et al. (2014) reported an even higher mean HbA1c of 7.9±1.4% in patients with diabetes, who did not show other comorbidities and had similar outcomes like non-diabetics [7]. The divergent results and heterogeneous patient groups suggest that more research in patients with diabetes undergoing TEER is warranted. Further studies are needed in order to evaluate if diabetes mellitus is a risk factor for worse outcome and therefore has to be considered more carefully in patient selection.

### Limitations

The main limitations are that this study was monocentric and that analyses were performed retrospectively. Echocardiographic follow-up was available just for 227 patients (73%) at one-year follow-up. Furthermore, diabetes duration and detailed characterization of microvascular complications have not been assessed. According to the mean HbA1c, diabetes was sufficiently controlled. It would be of interest, if patients with worse controlled diabetes and higher HbA1c show similar results. Therefore, multicenter and prospective studies with detailed characterization of patients with diabetes, who undergo TEER, are necessary with a long-term follow-up.

In our study, inclusion and exclusion criteria deviated from those of the randomized controlled landmark studies in TEER: following the recommendations by the German Society of Cardiology patients with severe primary and secondary MR were evaluated by a multidisciplinary team and suitability regarding mitral valve anatomy was judged by an experienced operator team [2, 3, 14]. Furthermore, a previous study could show that success of TEER improves with increasing operators’ experience [15]. Even though TEER at our institute was performed by experienced operators, it cannot be excluded that operators‘ experience may have affected outcome of TEER in our study.

## Conclusions

In a real-world setting, TEER seems to be similarly safe and effective in patients with diabetes and severe MR compared to those without diabetes with similar success of procedure and intrahospital mortality. Patients with diabetes were more diseased and had more comorbidities. Nevertheless, there was no difference regarding all-cause mortality, hospitalization due to heart failure, NYHA class or MR severity between both groups at one-year follow-up. Therefore, patients with diabetes and severe MR should not be excluded from TEER.
